# Pretemporal Transcavernous Approach for Resection of Non-meningeal Tumors of the Cavernous Sinus: Single Center Experience

**DOI:** 10.3389/fsurg.2022.810606

**Published:** 2022-02-17

**Authors:** Meng Huang, Jun Su, Qun Xiao, Qianquan Ma, Wenyong Long, Qing Liu

**Affiliations:** ^1^Department of Neurosurgery in Xiangya Hospital, Central South University, Changsha, China; ^2^Department of Neurosurgery in Hunan Children's Hospital, Changsha, China; ^3^Department of Neurosurgery in Peking University Third Hospital, Peking University, Beijing, China

**Keywords:** cavernous sinus, non-meningeal tumor, pretemporal transcavernous approach, microscopic surgery, surgical outcome

## Abstract

**Objectives:**

To study the outcomes of the pretemporal transcavernous approach in the treatment of non-meningeal tumors involving cavernous sinus and to investigate the surgical strategy for these lesions.

**Methods:**

We conducted a retrospective study of 45 patients with non-meningeal tumors involving cavernous sinus. All 45 patients received microsurgical resection *via* the pretemporal transcavernous approach from April 2012 to January 2019 by the same neurosurgeon. We analyzed clinical manifestations, image data, perioperative complications, surgical outcomes, functional outcomes, and follow-up data of these patients.

**Results:**

Gross total resection was achieved in 38 cases (84.4%) of the 45 patients. Preoperatively, a total of 64 individual cranial nerves were affected. Postoperatively, 92.2% of 64 impaired cranial nerves completely or partially restored function, 7.8% had worsened function compared with their preoperative statuses, and 5 new cranial nerve deficits (CNV) were observed in five patients during the last follow-up. Seven patients presented transient new cranial nerve deficits (5 CNIII and 2 CNVI), three cases suffered transient worsen cranial nerve deficits (3 CNIII and 1 CNVII). There were no cases of intracranial hematoma, intracranial infection, cerebrospinal fluid leaks, and death. The progression of residual tumor was observed in two patients (1 chordoma and 1 pituitary adenoma).

**Conclusions:**

Non-meningeal tumors involving cavernous sinus can be safely and radically removed with less morbidity and mortality. Pretemporal transcavernous approach is an ideal approach to the cavernous sinus and can be tailored individually.

## Introduction

The pathologies of the cavernous sinus (CS) tumors are various, including meningioma, pituitary adenoma, schwannoma, hemangioma, chordoma, chondroma, and so on. As the most common type of tumor of CS, meningioma has attracted the most attention from neurosurgeons. Due to the low total resection rate, high rate of complications and the good response to the radiotherapy, the enthusiasm for aggressive resection for meningioma has been tempered and more attention has been switched to protect functions of the cranial nerve (1). Compared to meningioma, the studies focusing on non-meningeal tumors of the CS are relatively rare. Besides, several studies found that the surgical outcome of non-meningeal tumors of CS is better, and cranial nerve morbidity is far less common than CS meningiomas (2, 3). Therefore, pursuing aggressive resection and preserving cranial nerve function in non-meningeal tumors of CS is more feasible than meningioma.

Since Parkinson's first reported a direct surgical approach to cavernous sinus, the CS is no longer a “no man's land.” Based on the pioneering anatomical work of Taptas and the surgical explorations by Parkinson's, Dolenc, and others, various extra or intradural surgical approaches have been developed to reach the CS and remove lesions (4, 5). To expand the exposure and reduce the retraction of brain, multiple invasive surgical approaches, such as the orbitozygomatic approach/extended middle fossa approach/transzygomatic middle fossa approach, are widely used in CS surgery (6–8). These approaches present several advantages, including a better operative trajectory with multidirectional access, a shallower surgical field, and less brain retraction through increased bone removal at the skull base. However, the invasive exposure *via* additional removal of skull base structure potentially results in cosmetic deformity and other complications (9, 10). Besides, ineffective exposure is common in CS surgery. Thus, a less invasive approach with tailored extent of bone removal, the exact exposure for an individual lesion, maintaining the advantages of the skull base approach, and reducing these risks are required for modern CS surgery. The pretemporal approach, being firstly introduced by Dr. De Oliveira in 1995 (11), is able to provide excellent exposure to the sellar, parasellar, interpeduncular regions, and the superior aspect of the petroclival region. Therefore, this approach was majorly considered by many surgeons as a practical way to treat basilar artery aneurysms (12). In this study, we describe our experience with the surgical management of non-meningeal tumors in CS *via* the pretemporal transcavernous approach and strategy to tailor the approach for individual neoplasm.

## Materials and Methods

### Patient Population

Between April 2012 and January 2019, a total of 45 patients with non-meningeal tumors involving the CS underwent microsurgical removal *via* pretemporal transcavernous approach. This study was approved by the Ethics Committee of Xiangya Hospital, Hunan, China, and written informed consent was obtained from all patients. All the protocols were performed following national guidelines. All surgeries were performed by our senior neurosurgeon (Dr. Qing Liu). This group of patients included 19 male and 26 female subjects and the mean age at the time of surgery in our patient population was 44.47 years (range 8–69 years).

### Inclusion Criteria

Confirmed by the histopathology, patients with CS non-meningeal tumors which included tumors primely originated from CS or secondarily invaded the CS and received microsurgery *via* pretemporal transcavernous approach were included in our study. Patients with CS meningioma or who underwent the surgery *via* other approaches were excluded.

### Radiological Evaluation and Classification

Preoperative magnetic resonance imaging (MRI) including scans with and without contrast was performed in every patient. For the lesions with skull base bone erosion or communicating tumors of the middle and posterior cranial fossa, the high-resolution computed tomography (HRCT) of the skull base was performed before the operation. Once the shifted/wrapped/narrowed intracavernous carotid were observed in preoperative MRI, the magnetic resonance angiography (MRA), or CT angiography (CTA) was performed before the surgery. The classification of trigeminal schwannoma was based on the criteria described by Jeong et al. (13), pituitary adenoma was graded according to the Knosp scale (14), and other tumors were classified based on the extension (Table 1).

**Table 1:** Characteristics of the study cohort.

**Case no**.	**Age/sex**	**Pathological diagnosis**	**classification/extension**	**Preoperative CN deficit**	**Craniotomy/approach**	**Tumor excision**
1	36/F	Epidermoid tumor	Intracavernous	–	FT + EDA	GTR
2	47/F	Epidermoid tumor	Intracavernous	–	FT + EDA	GTR
3	35/F	Epidermoid tumor	Intracavernous	V/VI	FT + EDA	GTR
4	27/M	Pituitary adenoma	Grade4	II/III	FT + EDA + SDA	partial removal
5	42/F	Pituitary adenoma	Grade4	–	FT + EDA + SDA	partial removal
6	53/F	Pituitary adenoma	Grade4	–	FT + EDA + SDA	GTR
7	50/F	Pituitary adenoma	Grade4	II	FT + EDA + SDA	partial removal
8	27/F	Pituitary adenoma	Grade4	II	FT + EDA + SDA	GTR
9	48/F	Pituitary adenoma	Grade4	II	FT + EDA + SDA	STR
10	35/M	Pituitary adenoma	Grade4	II	FT + EDA + SDA	GTR
11	62/M	Hemangioma	Intraorbital with CS extension	II/III/IV/V/VI	FTO + EDA	GTR
12	24/M	Hemangioma	Intraorbital with CS extension	II/III/V	FTO + EDA	GTR
13	25/M	Hemangioma	Intracavernous	II/III	FT + EDA	GTR
14	40/M	Hemangioma	Intracavernous	–	FT + EDA	GTR
15	40/M	Chordoma	CS, petroclival, supra-sellar	III/V/VI	FT + EDA	STR
16	30/F	Chondroma	M	II	FT + EDA	GTR
17	32/M	Schwannoma	Me3	–	FT + EDA	GTR
18	31/F	Schwannoma	Mpe3	II/III	FT + EDA	GTR
19	57/M	Schwannoma	MP	III/V	FT + EDA	GTR
20	58/M	Schwannoma	MP	II/VI/VIII	FT + EDA	STR
21	47/F	Schwannoma	Mpe3	II/V	FT + EDA	GTR
22	16/F	Schwannoma	MPe3	II	FT + EDA	GTR
23	67/F	Schwannoma	Me3	–	FT + EDA	GTR
24	32/F	Schwannoma	MP	V	FT + EDA	GTR
25	57/F	Schwannoma	MPe3	V/VII/VIII	FT + EDA	GTR
26	48/M	Schwannoma	M	II/VI	FT + EDA	GTR
27	46/M	Schwannoma	Me3	V	FT + EDA	GTR
28	63/F	Schwannoma	Me1,2,3	II	FT + EDA	GTR
29	40/F	Schwannoma	Me3	V	FT + EDA	GTR
30	8/M	Schwannoma	Me1	–	FTO + EDA	GTR
31	69/M	Schwannoma	Me1	–	FTO + EDA	GTR
32	66/M	Schwannoma	Mpe1	II/III/V/VI	FTO + EDA	GTR
33	48/F	Schwannoma	Me1	II/V	FTO + EDA	GTR
34	59/F	Schwannoma	M	II/III/IV/V/VI	FT + EDA	GTR
35	29/F	Schwannoma	E1m	II/III	FTO + EDA	GTR
36	43/F	Schwannoma	M	–	FT + EDA	GTR
37	35/M	Schwannoma	MP	II/III/IV/V	FT + EDA	GTR
38	56/F	Schwannoma	M	–	FT + EDA	GTR
39	62/M	Schwannoma	M	II	FT + EDA	GTR
40	45/F	Schwannoma	M	V	FT + EDA	GTR
41	65/M	Schwannoma	MP	–	FT + EDA	GTR
42	48/M	Schwannoma	Me3	V	FT + EDA	GTR
43	45/F	Schwannoma	M	V	FT + EDA	GTR
44	68/F	Schwannoma	ME1	II	FTO + EDA	GTR
45	40/F	Schwannoma	M	V	FT + EDA	GTR

### Surgical Technique and Strategies

Each patient was positioned supine and the head was fixed in tripoint headrest, rotated 35–45° to the opposite side, extended 10°, and inclined 5° in relation to the floor, and malar eminence was posed in the highest and central point of the operation field. A frontotemporal incision was made beginning at the superior border of the zygomatic arch, closing to the tragus. The incision then proceeded superiorly to the highest point of the auricle, then curved anteriorly to the end just behind the hairline until the midline was reached. After the cutaneous flap was reflected anteroinferiorly, interfacial dissection was performed until the orbital edge and temporal muscle were well-exposed. The temporalis muscle was dissected from the bone and reflected posteroinferiorly. Following the elevation of a frontotemporal bone flap, the bones of the temporal squama were removed to reach the level of the floor of the middle cranium fossa and the temporal facet of the sphenoid greater wing was removed to completely expose the polar of the temporal lobe. Then the sphenoid ridge was drilled extensively until the lateral limit of the superior orbital fissure was reached, and the posterior third of the lateral and superior orbital wall were shelled. If the tumor exhibited intra-orbital extension, the orbital osteotomy was performed. Then the meningo-orbital band was cut and the dura propria of the temporal lobe was peeled from the lateral wall of the cavernous sinus. And then the nerves that pass through the lateral wall of the cavernous sinus, including the oculomotor nerve, trochlear nerve, and the V1 and V2 branches of the trigeminal nerve, were identified as possible. The selective removal of the anterior clinoid process (ACP) depended on the need for the surgery. When necessary, the dura was cut in a curved T-shaped fashion with the vertical arm along the indentation of the sphenoid wing and sometimes might extend along the anterior petroclinoid ligament. The tumors were debulked and resected piece by piece and the part of tumors located in the cavernous sinus cavity were resected through the spaces between nerves. After surgery, the tumor cavity and the cavernous sinus were properly filled with the gelatin sponge and the dura was watertightly sutured. Since the bone of the skull base was not removed, the reconstruction of the skull base is unnecessary.

In most cases, once the superficial layer of the CS lateral wall was peeled from the deep layer, the tumor was encountered, then, it was debulked and removed as dissection proceeds. The triangles of the cavernous sinus were usually distorted due to the tumors and sometimes were difficult to recognize, so the space between two nerves was a safe area to incise.

#### Craniotomy

In our cohort, 37 (82.2%) of 45 craniotomies were performed frontotemporal (FT) approach, 8 (17.8%) were performed frontotemporal craniotomy combined with an orbital osteotomy (FTO) for tumors that involved the CS and the orbit at the same time.

## Results

### Clinical Features

A total of 45 patients with non-meningeal tumors of CS were enrolled in this study. The pathological type of tumors in this study include schwannoma (29, 64.4% of patients), pituitary adenoma (7, 15.6%), hemangioma (4, 8.9%), epidermoid tumor (3, 6.7%), chordoma (1, 2.2%), and chondroma (1, 2.2%). The presenting symptom in our patient cohorts included headache (42.2%), dizziness (17.8%), visual impairment (48.9%), diplopia (20.0%), ptosis (8.9%), facial numbness (28.9%), masseter weakness (8.9%), hearing disturbance (11.1%), menstrual disorder (6.7%), acromegaly (4.4%), exophthalmos (8.9%), hemiparesis (2.2%), and incidental finding (6.7%).

### Cranial Nerve Dysfunction

Preoperative cranial nerve dysfunction (CND) was observed in 34 patients (75.6%). The CNII dysfunction (48.9% of patients) was most common, followed by CNV (40.0%), CNIII (22.2%), CNVI (17.8%), CNIV (6.7%), CNVIII (4.4%), and CNVII (2.2%).

### Surgical Outcomes

Gross total resection (GTR) was achieved in 38 cases (84.4%), subtotal resection (STR) was achieved in four cases (8.9%), debulking or biopsy was achieved in three cases (6.7%). The gross total removal rate (GTR) was various in different pathological tumors. The GTR in hemangioma, epidermoid, and chondroma, were the highest (100%), followed by schwannoma (95.7%), pituitary adenoma (28.6%), chordoma (0%). For pituitary adenomas with partial removal (three patients) underwent the second surgery *via* transsphenoidal approach and the residual tumors within the sphenoid sinus were completely resected. The reason for STR included residual tumor was strictly adherent to the CNVII/VIII and brain stem, located in the opposite clival area, or invaded the bone of clival and dorsum sellae.

### Clinical Outcomes

There were no cases of intracranial hematoma, re-operation, tracheotomy, or death. Table 2 lists the cranial nerves involved preoperatively, postoperatively, and their function at the last followed up. Postoperatively, symptoms of cranial nerves were improved in 30 cases (46.9%), unchanged in 29 cases (45.3%), and worsened in five cases (7.8%). Five patients developed a new CNV deficit, seven patients suffered a transient new CNs deficit (CNIII and CNVI), three patients suffered a transient worsen CNs deficit (CNIII and CNVII).

**Table 2:** Preoperative and postoperative deficit of cranial nerves.

**Cranial nerve**	**Preoperative**	**Postoperative**			
		**Improved**	**Unchanged**	**Worse**	**New**
				**(transient)**	**(transient)**
**Pituitary adenoma**					
II	5	5	0	0	0
III	1	1	0	0 (1)	0 (2)
IV	0	0	0	0	0
V	0	0	0	0	0
VI	1	1	0	0	0
VII	0	0	0	0	0
VIII	0	0	0	0	0
Total	7	7	0	0	0
**Schwannoma**					
II	13	11	2	0	0
III	5	2	0	4 (1)	0 (3)
IV	2	0	2	0	0
V	14	3	10	1	4
VI	5	3	2	0	0 (2)
VII	1	0	1	0	1 (1)
VIII	2	1	1	0	0
Total	42	20	18	5	5
Others^*^					
II	4	2	2	0	0
III	4	1	0	0	0
IV	1	0	1	0	0
V	4	0	4	0	1 (0)
VI	2	0	2	0	0
VII	0	0	0	0	0
VIII	0	0	0	0	0
Total	15	3	9	0	1
**All**					
II	22	18	4	0	0
III	10	4	0	4 (2)	0 (5)
IV	3	0	3	0	0
V	18	3	14	1	5
VI	8	4	4	0	0 (2)
VII	1	0	1	0	1 (1)
VIII	2	1	1	0	0
Total	64	30 (46.9%)	29 (45.3%)	5 (7.8%)	6

* *Including epidermoid tumor, hemangioma, chordoma, and chondroma*.

### Follow-Up Outcomes

Mean follow-up was 51.3 ± 24.1 months in our series. No tumor recurrence was observed following total resection during the follow-up period. Three pituitary adenoma patients with partial removal underwent transsphenoidal surgery 1 month after the first surgery and no recurrence was observed during the follow-up period. In two patients who underwent STR (chordoma and pituitary adenoma), tumor progression was observed in the 6th month after gamma knife treatment. The residual tumors of the other two patients who underwent STR were well-controlled by the gamma knife treatment.

### Illustrative Cases

#### Case 1

**Figure 1:**
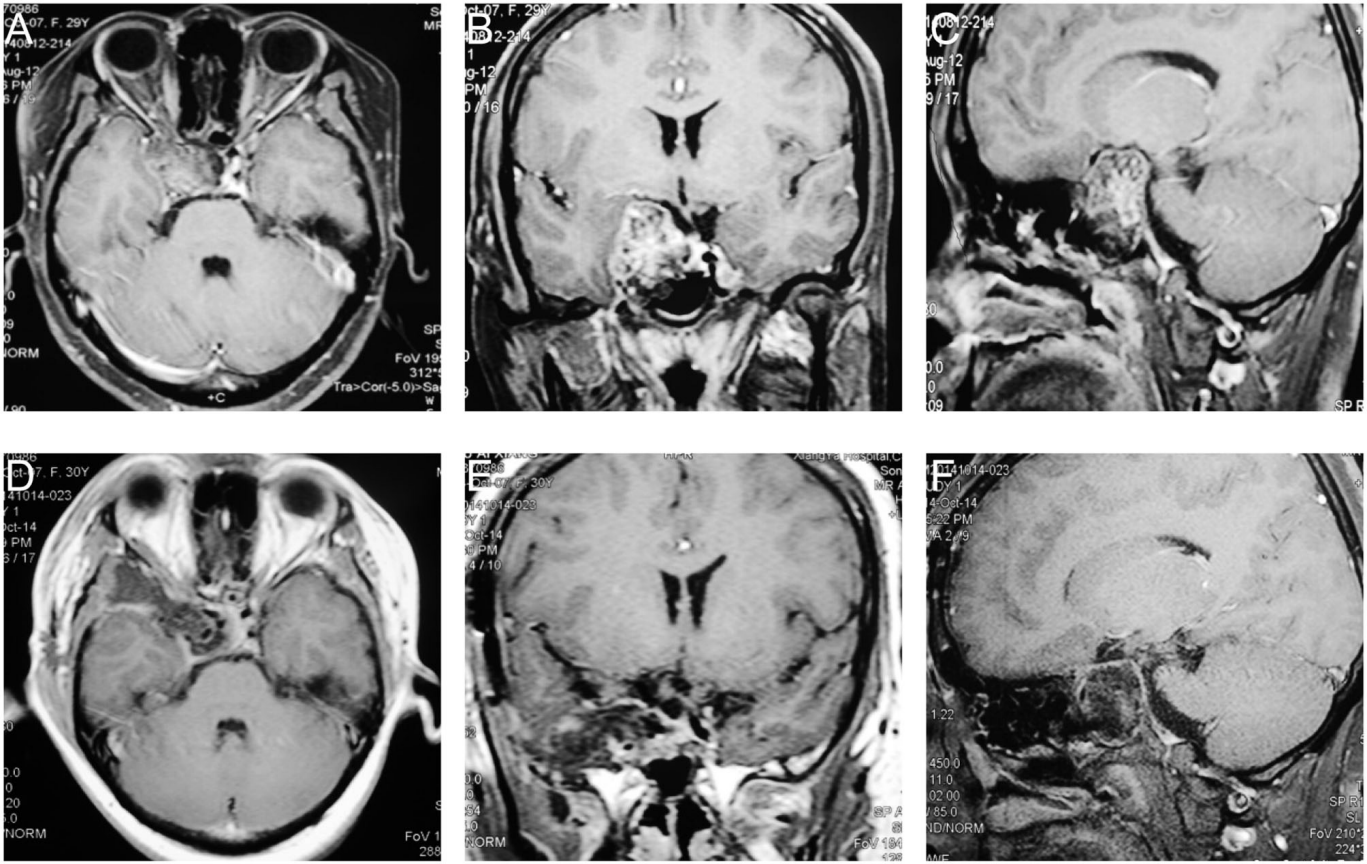
The preoperative and postoperative MRI imaging of case 1. **(A–C)** Preoperative MRI showed the tumor involved intrasellar and the right CS. **(D–F)** Postoperative MRI showed the tumor was gross total resected.

A 30-year old female patient, her clinical symptoms included Hypometropia, amenorrhea, and lactation. Eight months ago, this patient underwent an operation *via* microscopic transnasal transsphenoidal approach and partial tumor remained and treated by gamma knife. However, the residual tumor progressed during the follow-up, MRI study showed a mass with equal T1 and equal T2 signal within slice shot T1 signal, located in the right CS. Enhanced MRI showed the enhancement of tumor is Heterogeneous (Figures 1A–C). Then the patient underwent microscopic surgery via pretemporal transcavernous approach using FT craniotomy. The tumor was gross total removed extradurally (Figures 1D–F). After the surgery, the patient did not suffer any surgery-related complications. The postoperative diagnosis was chondroma.

#### Case 2

**Figure 2:**
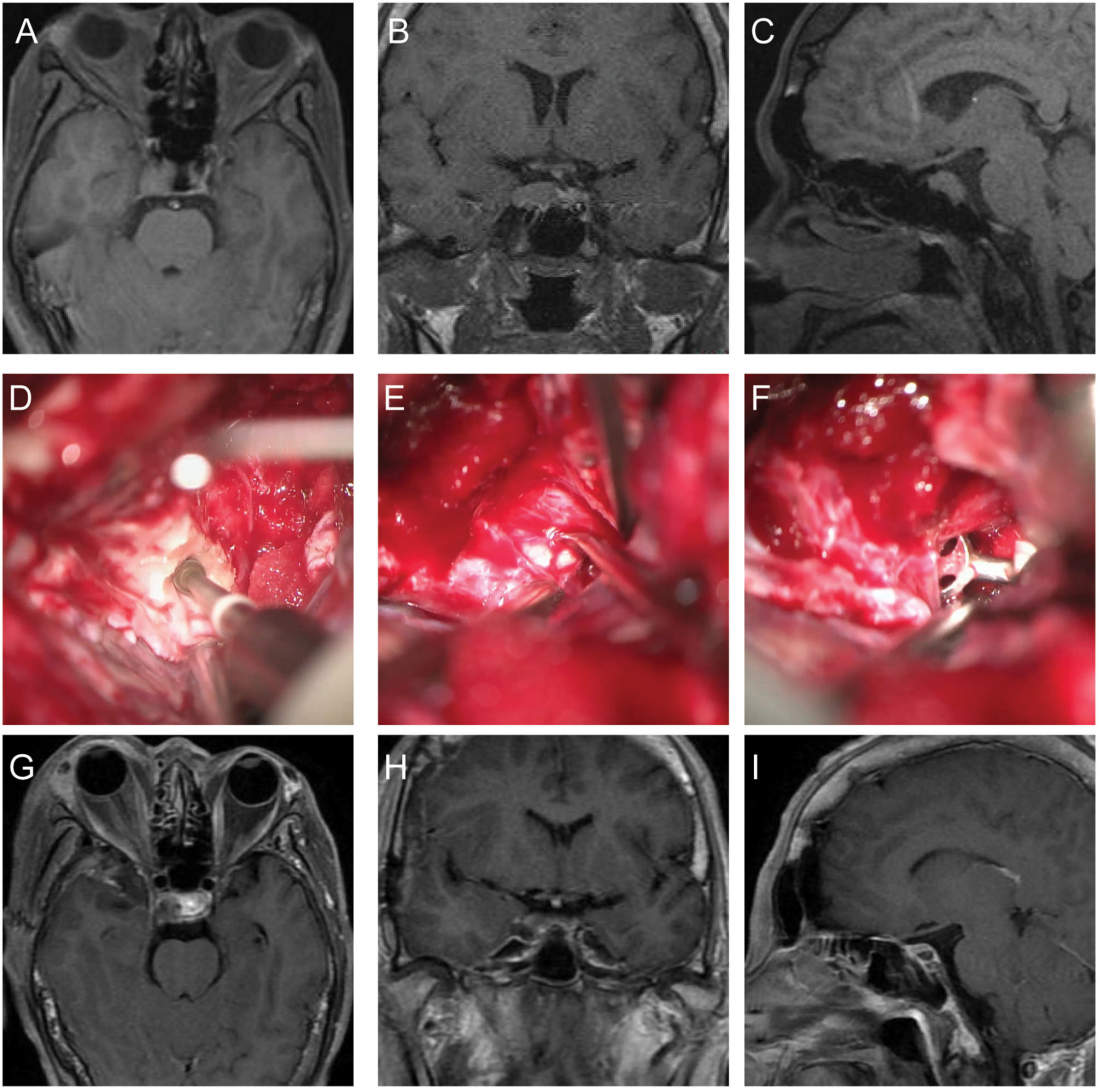
The preoperative and follow-up MRI imaging and intraoperative photography of case 2. **(A–C)** Preoperative MRI showed the tumor located on the right side of sella and invaded the post-superior part of the right CS. **(D)** Drilling the ACP extradurally under microscope. **(E)** Exposure of tumor through the Parkinson's triangle. **(F)** Removal of the tumor using microcurette. **(G–I)** Follow-up MRI showed the original tumor was completely resected without signs of residue or recurrence.

A 53-year old female patient had clinical features of acromegaly and was diagnosed with diabetes. She did not have other symptoms. MRI study showed a mass with slightly long T1 and slightly short T2 signal located on the right side of sella and invaded the right CS. Enhanced MRI showed the enhancement of tumor is weaker than normal pituitary (Figures 2A–C). The patient underwent a microscopic operation *via* the pretemporal transcavernous approach using FT craniotomy. Due to the tumor located in the post-superior part of CS, the combining extradural and subdural approach was used. The ACP was drilled extradurally (Figure 2D). After cutting the roof of CS, the superficial layer of the CS lateral wall was further peeled away from the deep layer. The tumor was detected and completely removed through the Parkinson's triangle (Figures 2E,F) and the pathological diagnosis was pituitary adenoma. After the surgery, the patient suffered a transient oculomotor palsy which recovered after 1 month. The last follow-up MRI study did not show any signs of recurrence (Figures 2G–I). The postoperative diagnosis was pituitary adenoma.

#### Case 3

**Figure 3:**
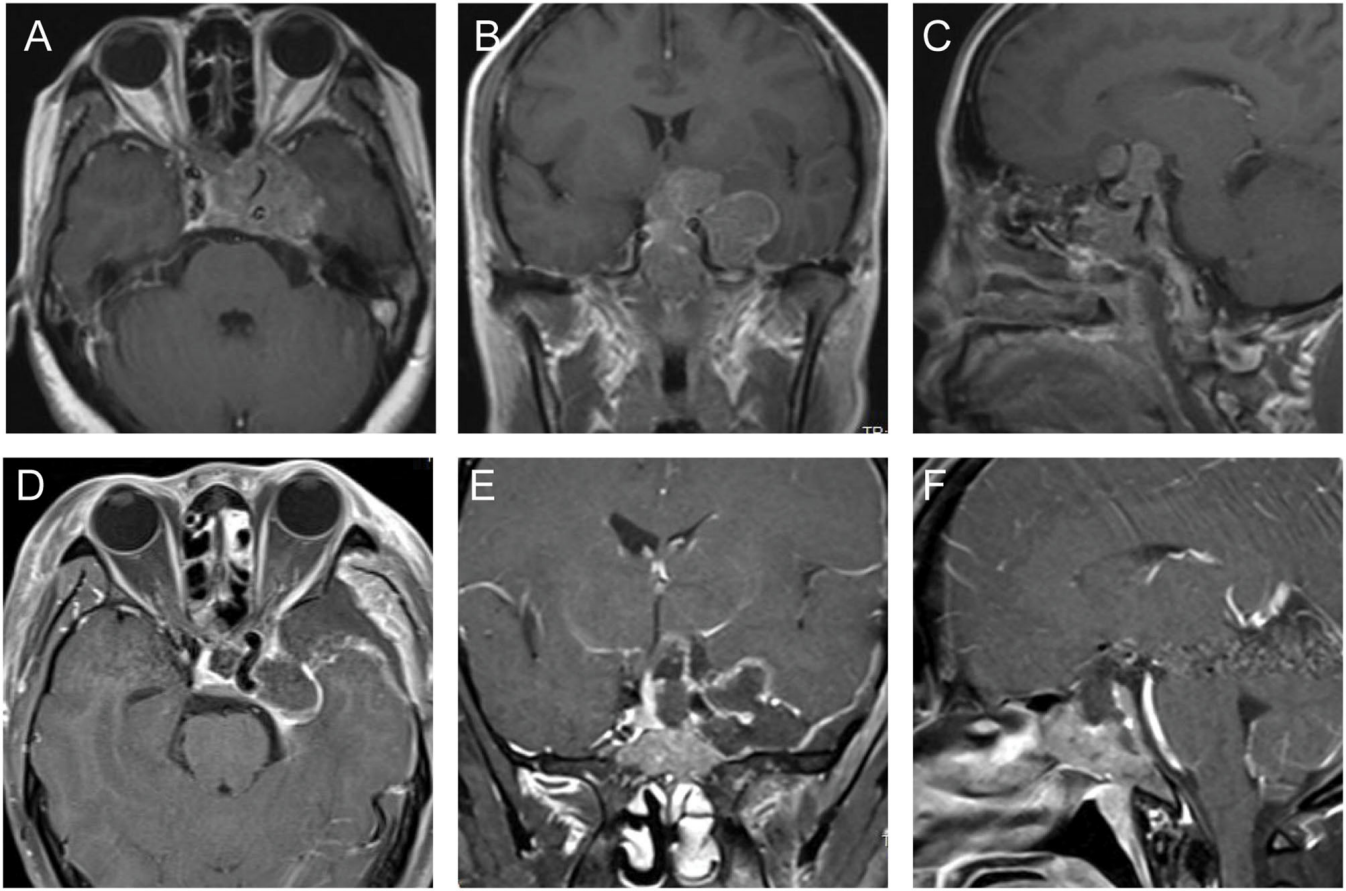
The preoperative and postoperative MRI imaging of case 3. **(A–C)** Preoperative MRI showed the tumor involved intrasellar, suprasellar region, sphenoid sinus, and the left CS. The intracavernous ICA was completely encapsulated by this tumor. **(D–F)** Postoperative MRI showed the most of the tumor was resected and part of the tumor remained in the sphenoid sinus.

This 42-year old female patient had presented with intermittent headache for several months. Cranial MRI demonstrated the presence of a sellar tumor, which extended toward the sphenoid sinus, suprasellar region, and the left CS with encapsulation of intracavernous ICA (Figures 3A–C). The preoperative diagnosis is a pituitary adenoma. The CS was approached *via* FT craniotomy and exposed extradurally. For further exposure and resection of the tumor in the intrasellar and suprasellar region and the posterior part of CS, the dura was cut in a curved T-shaped fashion. After peeling off the superior layer of the lateral wall of CS, the temporal lobe was retracted laterally together with the superior layer of the lateral wall of CS. Part of the tumor involved in the CS was exposed and resected/sucked completely through the space between nerves. The suprasellar tumor was resected via the space between ICA and optic nerve and the intrasellar tumor was sucked and scraped. Considering the risk of postoperative cerebrospinal fluid leakage and intracranial infection, only the tumor extended in the sphenoid sinus was left to second-stage surgery (Figures 3D–F). One month later, the residual tumor was completely removed via transsphenoidal surgery. This patient did not develop any new cranial nerve paralysis. The postoperative diagnosis was pituitary adenoma.

#### Case 4

**Figure 4:**
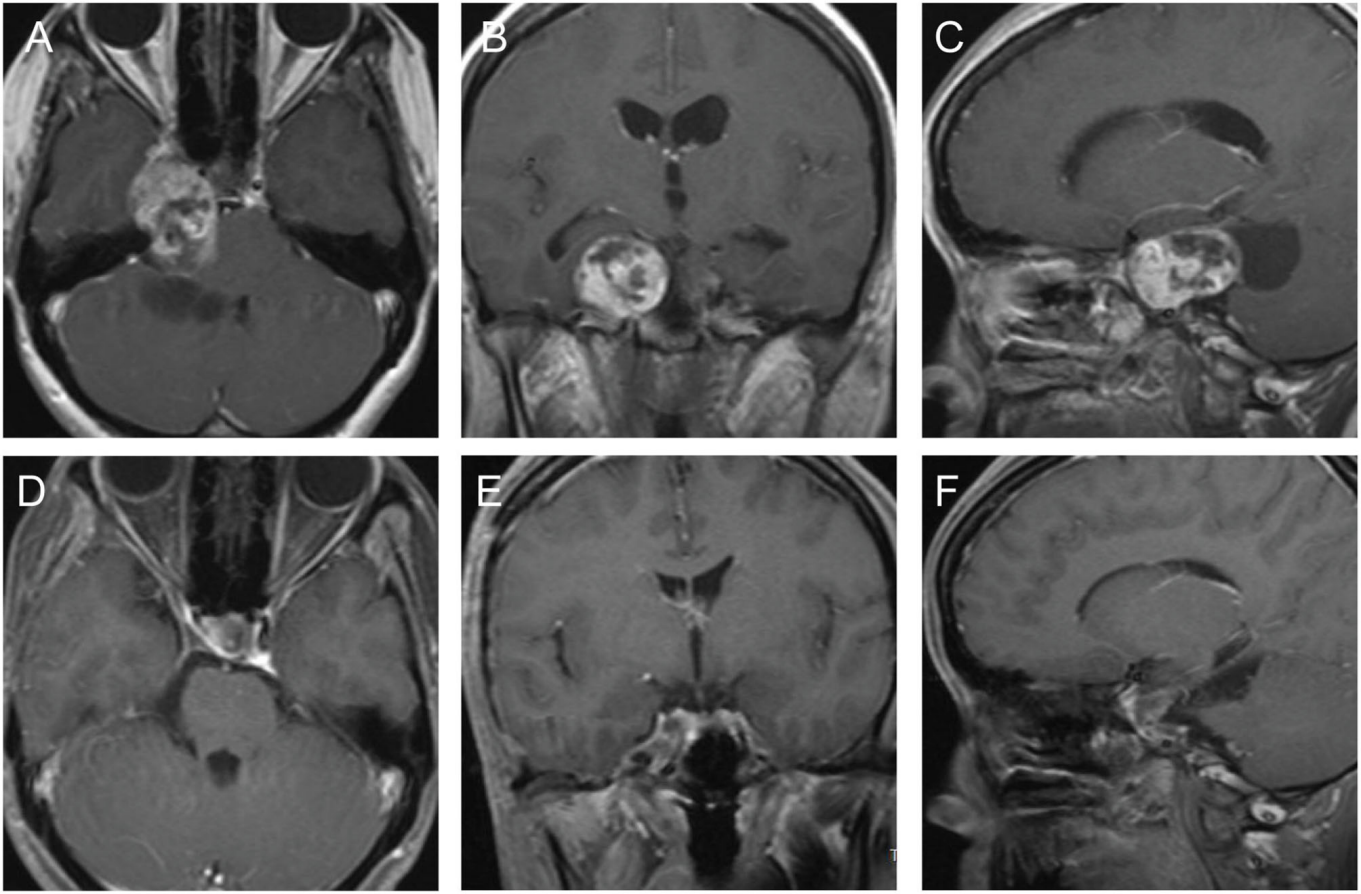
The preoperative and postoperative MRI imaging of case 4. **(A–C)** Preoperative MRI showed a dumbbell-shaped tumor involving both the right middle and posterior fossae. **(D–F)** Postoperative MRI showed the most of the tumor was completely removed.

This 32-year old female patient suffered from intermittently right facial pain for 4 years, and dizziness for 2 weeks. Preoperative MRI documented an enhancing dumbbell-shaped tumor that involved both the right middle and posterior fossae (Figures 4A–C). The patient underwent a microscopic operation via the pretemporal transcavernous approach. The FT craniotomy was performed and the cavernous was approached extradurally. The tumor was debulked and resected piece by piece. The bone of the apical petrous was destructed by the tumor, which provides “natural” access to remove the part of the tumor located in the posterior fossa. The tumor was completely removed (Figures 4D–F). After the surgery, the facial pain was improved and the patient did not suffer any surgery-related complications. The postoperative diagnosis was schwannoma.

#### Case 5

**Figure 5:**
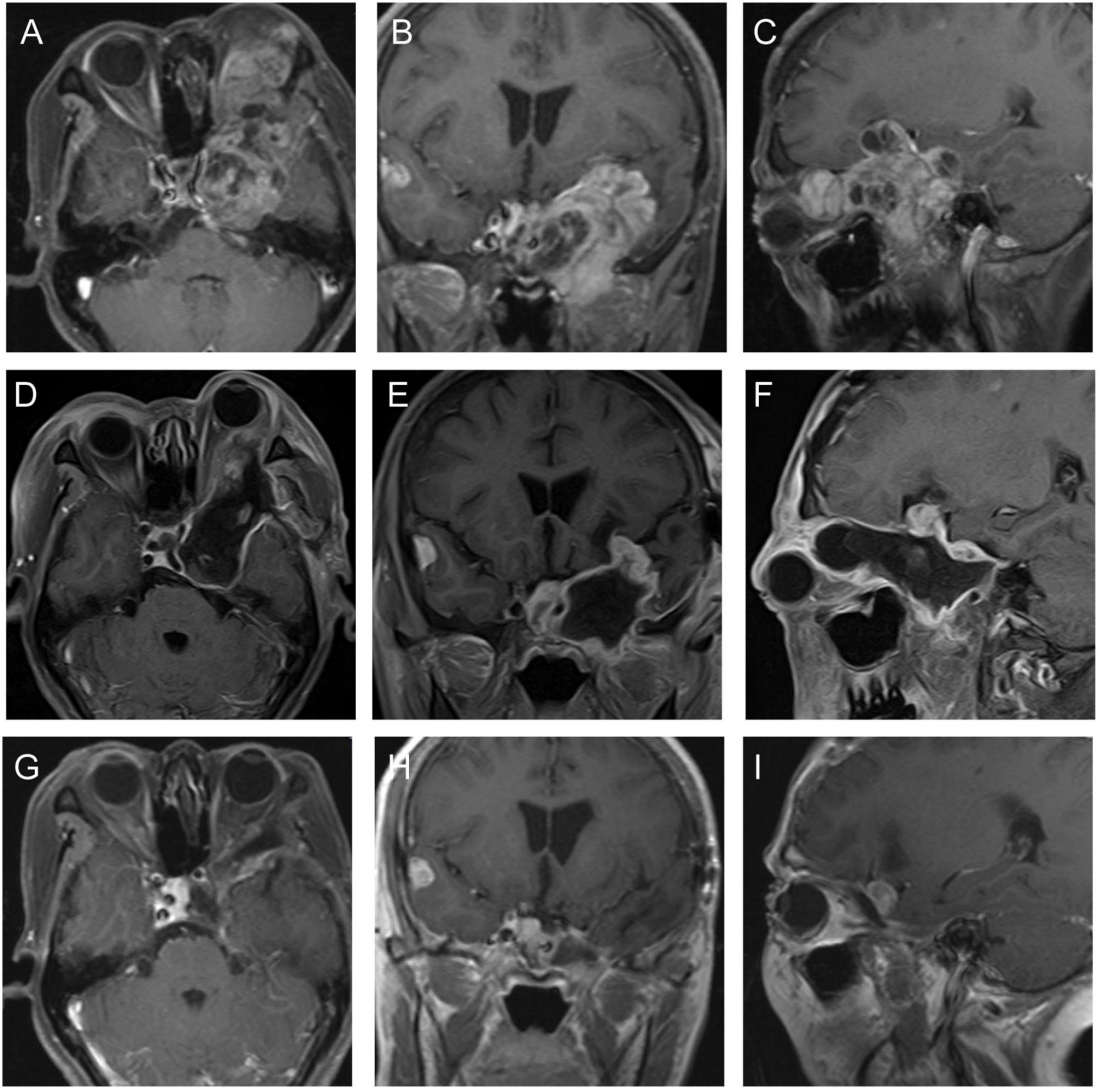
The preoperative, postoperative, and follow-up MRI imaging of case 5. **(A–C)** Preoperative MRI showed the trigeminal schwannoma involved the left middle fossa, orbit, and infratemporal fossa. **(D–F)** Postoperative MRI showed the most of the tumor was completely removed. We considered the postoperative dura enhancement was due to the scarring formation from the primary surgery. **(G–I)** Follow-up MRI showed no recurrence.

This case was a recurrent trigeminal schwannoma. Three years ago, this patient underwent a microscopic operation for trigeminal schwannoma limited in left middle fossae via subdural approach. In this admission, preoperative MRI documented an enhancing larger tumor involving middle fossae, orbital, and subtemporal fossae (Figures 5A–C). The patient underwent a microscopic surgery *via* the pretemporal transcavernous approach. The FTO craniotomy was performed and the cavernous was approached extradurally. The tumor was debulked and resected piece by piece. The tumor located in the subtemporal fossae was removed through the enlarged ovale foramen. The tumor was completely removed (Figures 5D–F). After the surgery, the patient only complained of hypopsia. The last follow-up MRI study did not show any signs of recurrence (Figures 5G–I). The postoperative diagnosis was schwannoma.

## Discussion

Surgical resection of tumors involved in CS is still a challenge for most neurosurgeons. Controlling bleeding and avoiding the injury of CN and ICA are the eternal topics in CS surgery. Compared with meningioma, removing the non-meningeal tumors involving in CS is relatively safe, and total resection is more achievable (2, 3, 15). These tumors differ from meningiomas in origination, invasion pattern, and consistence. The displacement is more common than encapsulation of ICA. Even encapsulated by non-meningeal tumors, the ICA can be easily dissected from these tumors, especially the pituitary adenomas, attributed to their relatively soft character. Thus, the exposure and control of petrous ICA and clinoidal ICA are unnecessary in most cases. In addition, ineffective exposure exists in CS surgery *via* invasive skull base approaches and may induce potential complications, such as cosmetic deformity, which are associated with this additional exposure (9, 16, 17). Therefore, it is possible and necessary to determine a less invasive approach for patients with non-meningeal tumors of CS. In this study, we focus on the operative techniques and surgical strategy for non-meningeal tumors of CS.

### Pretemporal Craniotomy Is an Ideal Approach to the Cavernous Sinus

The pretemporal approach was firstly described by de Oliveira and combined the advantage of pterional, subtemporal, and temporopolar approaches in one craniotomy (11, 18). Different from OZ craniotomy, which was widely used in the skull basal approach, pretemporal craniotomy retained the rim of the orbital and the zygomatic arch to reduce some surgical complications. Removal of the squamous part of the temporal bone to reach the level of the skull base and temporal facet of the sphenoid greater wing are extremely important to expose the entire temporal lobe including the temporal polar. Due to the blockage of the zygomatic arch, the temporalis could not be pulled down sufficiently, which might cause the so-called “threshold effect” and block the operative field. Krisht and Kadri (19) reported that drilling the superior aspect of the zygomatic arch at the level of the zygomatic notch could help to achieve inferior reflection of the temporalis muscle similar to what is achieved with the OZ approach. In this study, we found that retracting the temporalis posterior-inferiorly could effectively relieve the blockage of the temporalis. Removal of the sphenoid wing to reach the lateral limit of the superior orbital fissure was indispensable for CS surgery, after which the orbital-meningeal band, the start point of transcavernous procedure, was exposed. The cavernous sinus is a pyramidal structure with an apex toward the SOF, the orbital bone might partially block the anterolateral view of CS during the surgery. In pretemporal craniotomy, the roof and lateral wall of the orbital were partially removed and the orbital rim was preserved, which can effectively avoid the postoperative enophthalmos (11, 19). We found that skeletonizing the superior and lateral walls of the orbital was enough for tumors without orbital extension and removing the posterior part of orbital walls was sufficient for exposing the orbital part of the tumor. The OZ approach, being advocated by many skull base surgeons for the access to the cavernous sinus, becomes necessary when the lesions extend into the 3rd ventricle, high AcomA aneurysms, and high basilar tip aneurysms. There is no need to perform the surgery with OZ approach when dealing with tumors in the CS which is located in the middle fossa. Therefore, the pretemporal approach is a “correctly” invasive option for approaches to the cavernous sinus when compared with the OZ approach.

### Completely Extradural Approach or Combined Extradural and Intradural Approach

Before the 1980s, the transcranial approaches for the cavernous sinus were intradural or subdural, then CS was accessed through the Parkinson's triangle or the roof of the CS. The traditional intradural approach has some disadvantages, including direct brain retraction, the sacrifice of temporal pole bridging veins, difficulty to identify cranial nerves. Based on the pioneering study of Taptas (20), Dolenc (21), and Umansky and Nathan (22) et al. in CS, the understanding of the CS is constantly improving. The great findings include that CS, located in the middle fossae extradural structure, is composed of venous plexus and the lateral of the CS wall have at less two layers, and the superficial layer can be peeled off from the deep layer easily (22–24). Then many extradural approaches have been reported (25). Compared to the intradural approach, the extradural approach has many advantages (25): fully exposure of the CS, easy identification of the cranium nerves that pass through the CS lateral wall, excellent control of the intracavernous carotid artery, extradural extraction of the brain, preserving of the sylvian vein, bridging vein and sphenoparietal sinus, good control of bleeding, and decreasing the CSF leakage. Most CS tumors without intradural extension can be removed extradurally by pretemporal transcavernous approach (case 1). However, in order to expose the entire CS, especially the posterior part of CS, subtemporal access is needed in extradural approaches, which make it necessary to remove the zygomatic arch and additional skull base bones (25). On the other hand, not all tumors are confined within CS and the subdural approach is unavoidable for resection of the subdural portion of tumors involving CS. Therefore, pure extradural approaches are not sufficient for all cases. In this study, we found that the combined extra- and intradural approach is more suitable than the extradural approach for tumors limited in the superior posterior part of CS and tumors with subdural extension. The combined approach remains the key advantages of the extradural approach, such as easy identification of the cranium nerves, extradural extraction of the brain, and good control of bleeding. Besides, the combined approach makes it easy to expose the posterior part of CS. After cutting the roof of CS intradurally, the out layer of the lateral wall of CS can be peeled completely, then the temporal lobe with the superficial layer of the lateral wall of CS can be retracted posterolaterally to expose the supra-posterior part of the CS sufficiently without performing zygomatic osteotomy (case 2). In addition, intradural steps can expose the areas adjacent to CS and release CSF to reduce intracranial pressure, which makes it relatively easy to expose the CS.

### Individualized Anterior Clinoidectomy

Anterior clinoidectomy, which is commonly applied in CS surgery (21, 24, 25), provides the exposure of the anterior part of the CS roof and the clinoidal segment of ICA, benefits to the control of distal ICA, and releases the ICA to reach the anterior pontine area. However, the removal of the anterior clinoid process may bring some risks, including CSF leakage, especially in cases with ACP pneumatization and neurovascular injury. Different from vascular disease, anterior clinoidectomy is not necessary for all kinds of CS tumors dissection. We found that the anterior clinoidectomy is unnecessary for most non-meningeal tumors in CS. The trigeminal schwannoma and invasive pituitary adenoma are the most common non-meningeal tumors in CS (2). Trigeminal schwannoma belongs to the intradural tumor (2) and rarely encapsulates the ICA. A pituitary adenoma may invade the CS through the medial wall and encapsulate the ICA, however, it can be removed easily because of its soft texture. With the popularity of MRI, CTA, and MRA, which provide the information about the tumor growth pattern, the relationship between the tumor and ICA (wrapped or simply shoved), the surgeon can make a judgment whether the control of the clinoidal ICA is required or not. We consider removing ACP is necessary for the following situations: ICA is completely wrapped with a high risk of injury during surgery; tumors extend to the orbital through the optic canal or internal part of the superior orbital fissure. In addition, it is useful to remove ACP for the surgery of small tumors confined in CS (case 2). After removing ACP, the surgeon can identify the CNIII/IV and select a suitable triangle to resect these tumors. In contrast, it is usually unnecessary to perform anterior clinoidectomy for larger tumors due to they often break through the deep layer of the lateral wall of CS, which provides a “natural” route to reach CS.

### Tumor Exposure in the Adjacent Region of CS

The CS tumors may involve the adjacent regions, including orbit anteriorly, sellar region medially, infratemporal fossa inferiorly, and petroclival region posteriorly. During the surgery of CS, these adjacent regions should be exposed. The intra-orbital region can be exposed after removing the lateral and superior wall of the orbital (case 5). If the tumor extended through the medial part of SOF or the optic canal, the ACP should be resected simultaneously. The sellar region can be reached via combining the traditional pterion approach (case 3). Infratemporal fossa and petroclival region extension are more common in trigeminal schwannoma and chordoma than other non-meningeal tumors. Both schwannoma and chordoma present the feature of bony erosion, which supports a “natural” corridor to the infratemporal fossa or posterior fossa (case 4). These adjacent areas should be exposed individually. The preoperative valuation is paramount for planning CS surgery (26). With the advance of neuroimaging, preoperative MIR, CTA, and HRCT can provide important information, such as the extent of the tumor, the relationship between the tumor and the ICA, the possible origination of the tumor, the possible pathological type, and the destruction of the skull base. Combining preoperative imaging and intraoperative findings, a neurosurgeon can design and tailor the approach from craniotomy to closure, to gain excellent exposure of the tumor and maximum resection.

## Conclusion

Non-meningeal tumors involving cavernous sinus can be safely and radically removed with less morbidity and mortality. Pretemporal transcavernous approach is an ideal approach to the cavernous sinus and can be tailored individually.

## Data Availability Statement

The original contributions presented in the study are included in the article/supplementary material, further inquiries can be directed to the corresponding author/s.

## Ethics Statement

The studies involving human participants were reviewed and approved by the Ethics Committee of Xiangya Hospital. Written informed consent to participate in this study was provided by the participants' legal guardian/next of kin.

## Author Contributions

MH, JS, and QL: conception and design. QX and QM: collection of data. MH and JS: analysis, interpretation of data, and drafting the manuscript. WL and QL: study supervision. All authors did the surgery procedure, revised the article, and all contributors to this study are included in the list of authors.

## Conflict of Interest

The authors declare that the research was conducted in the absence of any commercial or financial relationships that could be construed as a potential conflict of interest.

## Publisher's Note

All claims expressed in this article are solely those of the authors and do not necessarily represent those of their affiliated organizations, or those of the publisher, the editors and the reviewers. Any product that may be evaluated in this article, or claim that may be made by its manufacturer, is not guaranteed or endorsed by the publisher.
